# The interplay between mothers’ and children behavioral and psychological factors during COVID-19: an Italian study

**DOI:** 10.1007/s00787-020-01631-3

**Published:** 2020-08-31

**Authors:** Elisa Di Giorgio, Daniela Di Riso, Giovanna Mioni, Nicola Cellini

**Affiliations:** 1 Department of Developmental Psychology and Socialization, University of Padova, Via Venezia 8, 35131 Padua, Italy; 2 Department of General Psychology, University of Padova, Padua, Italy; 3 Department of Biomedical Sciences, University of Padova, Padua, Italy; 4 Padova Neuroscience Center, University of Padova, Padua, Italy; 5 Human Inspired Technology Center, University of Padova, Padua, Italy

**Keywords:** COVID-19 outbreak, Mothers and children, Home confinement, Work condition

## Abstract

Italy has been the first nation outside of Asia to face the COVID-19 outbreak. To limit viral transmission of infection, by March 10th, 2020, the Italian Government has ordered a national lockdown, which established home confinement, home (*smart*) working, and temporary closure of non-essential businesses and schools. The present study investigated how these restrictive measures impacted mothers and their pre-school children’s behavioral habits (i.e., sleep timing and quality, subjective time experience) and psychological well-being (i.e., emotion regulation, self-regulation capacity). An online survey was administered to 245 mothers with pre-school children (from 2 to 5 years). Mothers were asked to fill the survey thinking both on their habits, behaviors, and emotions and on those of their children during the quarantine, and retrospectively, before the national lockdown (i.e., in late February). A general worsening of sleep quality and distortion of time experience in both mothers and children, as well as increasing emotional symptoms and self-regulation difficulties in children, was observed. Moreover, even when the interplay between the behavioral and psychological factors was investigated, the factor that seems to mostly impact both mothers' and children's psychological well-being was their sleep quality. Overall, central institutions urgently need to implementing special programs for families, including not only psychological support to sustain families with working parents and ameliorating children's management.

**Electronic supplementary material:**

The online version of this article (10.1007/s00787-020-01631-3) contains supplementary material, which is available to authorized users.

## Introduction

An outbreak of 2019 novel coronavirus disease (COVID-19) in Wuhan, China, has spread quickly worldwide, forcing the World Health Organization (WHO) to declare first COVID-19 as a public health emergency of international concern (30 January 2020), and then to define the situation as a pandemic (11 March 2020) [1].

Italy has been the first nation outside of Asia to face the COVID-19 outbreak. By May 1st, Italy had 204.576 confirmed cases, with 26.049 deaths, according to the Istituto Superiore di Sanità (“the COVID-19 Task force of the Department of Infectious Diseases and the IT Service Istituto Superiore di Sanità”, https://www.iss.it/). To limit viral transmission of COVID-19 infection, by March 10th, 2020, the Italian Government has ordered a national lockdown, expanding the strict domestic quarantine policies already imposed in late February for few Northern cities (Decree of the President of the Council of Ministers). The national lockdown establishes a range of severe disease control strategies including movement restriction, home (*smart*) working, and temporary closure of non-essential businesses and schools of every order and degree. Even though these measures are extremely important in preventing additional spreading of the coronavirus infection, this prolonged home confinement might affect people's mental health [2].

Studies that investigated the impact of the quarantine adopted in *severe acute respiratory syndrome* (SARS) and *Middle-east respiratory syndrome* (MERS) containment on mental health, reported a high prevalence of both psychological symptoms, such as depression, low mood, irritability, anger emotional disturbance, anxiety [3–5], and behavioral symptoms, such as poor sleep quality, sleep disturbance, and insomnia [6]. As regards COVID-19, current literature about this issue is still updating, with few papers published up to date [2, 7–9].

In the COVID-19 outbreak, families and children are particularly vulnerable, accounting for negative effects on their physical and psychological well-being [2, 10]. The quarantine is forcing a protracted cohabitation of the family members that could also be detrimental to the emotional climate. Broadly speaking, for parents, the boundaries between work and home have completely broken down. Family needs to completely rearrange children's management due to school closure. Moreover, mothers who continue to work at home in a *smart modality*, struggle to maintain a balance between work and family, presumably accounting for the highest level of psychological distress.

As for children, extended school closure might interfere with healthy habits, regarding outdoor activity and daylight exposure, diet, sleep, and with psychological equilibrium due to boredom, lack of direct information about the disease outbreak, and total absence of in-person relationships with peers [11]. As Sprang and Silman [12] reported, about 30% of children quarantined during pandemic diseases might meet post-traumatic Stress Disorder (PTSD) criteria. Evidence suggested that one of the most protective factors in preserving both physical and psychological well-being in children is having a structured and pre-planned day, facilitated during school time (*Structured Days Hypothesis*, SDH) [13].

Considering the current state, a timely understanding of how the restrictions, and the imposed home confinement impact mothers' and children's behavioral habits and psychological well-being is urgent. Therefore, the present research aims at (a) characterizing the changes in mothers' and children’ sleep quality, subjective time experience, emotional symptoms, and self-regulation capacity during the lockdown compared to the period immediately before; (b) describing the interplay between behavioral and psychological factors associated with mothers and children difficulties. Furthermore, we will take into account the different mothers’ working conditions during the lockdown.

In line with previous studies on the negative effects of quarantine [2, 11, 12], we expected to observe during the lockdown a general worsening of sleep quality and distortion of time experience in both mothers and children, as well as increasing emotional symptoms and self-regulation difficulties in children. Moreover, we expected that changes in mothers’ and children's sleep quality, as well as in the management of daily routines, would determine a worsening in children' self-regulation capacity and negatively impact mothers’ emotional symptoms [14].

## Methods

### Participants and procedure

An online survey, that involved mothers and their children on the national territory, was administered from April 1st to April 9th, 2020. Participants were recruited through online advertisements on research-related websites and social media groups. Inclusion criteria were: (a) being at least 18 years old, and (b) having children between 2 and 5 years of age. Pre-school children were selected because they need more maternal attention compared to school-aged children. A total of 256 respondents began the survey. The final sample size was 245 participants (*M*_age_ = 37.31 years, SD_age_ = 4.61, age-range = 23–49 years). The mean age of children (females = 117) was 4.10 years (SD_age_ = 0.92).

After the respondents had provided informed consent, they responded to some general questions concerning socio-demographic characteristics including gender, age, education, and employment, as well as some questions related to the COVID-19 infection (e.g., how much they feel scared by the COVID-19, where they obtain information about the virus, whether they know people infected by or deceased due to COVID-19). Then, mothers were asked to fill the survey thinking both on their habits, behaviors, and emotions and on those of their children. Importantly, they have to respond to the questions relative to their lives in the present, during the quarantine (from 1st to the 9th of April, after three weeks of confinement), and retrospectively to the week before the national lockdown (i.e., last week of February from 24 to 29th of February). The project will include also a follow-up, in which mothers will fill the survey at the end of the emergency to control for psychological changes after lockdown. The survey took about 30 min to be completed. The project was approved by the Ethical Committee of Psychological Research of the University of Padova (Prot. no.: 3521).

## Measures

### Behavioral factors

#### Sleep quality

Mothers’ sleep quality was assessed using the total score of the Pittsburgh Sleep Quality Index (PSQI) [15] in its Italian version [16], a valid, reliable, and widely used self-report questionnaire. The range of this 18-item scale is from 0 to 21, with higher scores indicating worse sleep quality. A score higher than 5 indicate poor sleep quality. From two questions of the same questionnaires, the average bedtime (at what time they went to bed to sleep, hh:mm) and waketime (at what time they wake up in the morning, hh:mm) of the mothers were extracted.

Children’s sleep quality was assessed using the total score of the Sleep Disturbance Scale for Children (SDSC, Italian version provided by Bruni) [17]. This 26-item scale has a score ranging from 26 to 130, with higher scores indicating greater sleep difficulties. A score higher than 39 can be considered a cut-off for identifying children with disturbed sleep. We also asked parents to report the average bedtime (at what time they went to bed to sleep, hh:mm) and waketime (at what time they wake up in the morning, hh:mm) of their children.

#### Time perception

To test the mothers’ subjective feelings of time, 13 items from the Italian adapted version of the Subjective Time Questionnaire were used (STQ) [18, 19]. The questionnaire contains several parts consisting of questions concerning (1) the typical (everyday) experience of the passage of time, (2) a retrospective look at long past time intervals, (3) subjective feeling of time, and (4) metaphors of time. To the purpose of the present study the items concerning the dimension of “metaphors of time” were not included. (1) The two questions that cover the perception of the present time reflect a more consistent view of the passage of time, i.e., state-like momentary perception of time. (2) In the second set of questions assessing retrospective judgments of longer time intervals and life periods, we selected the item that asked how fast the week has passed (first week of February, and last week). (3) The third set of questions consists of statements on the subjective experience of time (anchors: 1 = strong rejection and 5 = strong approval) that refer to the feeling of time pressure/time compression (5 statements, e.g., "I often think that time is running out.") or to the feeling of time expansion/time affluence (5 statements, e.g., "My time is not filled.").

For children, three items were included to test the subjective feeling of time and time management. Two out of three items were selected from Porcelli et al. [20] “He/she does not experience that time has passed (in Italian: “Non si accorgeva che passava il tempo”) and “He/she doesn’t respect the routine” (in Italian: “Non seguiva le routine stabilite”). We included the third item to investigate children’ subjective perception of boredom which is strongly correlated with time [21]: “Is he/she experiencing boredom” (in Italian: “Si sentiva annoiato/a”).

### Psychological factors

#### Executive functions

The Behavior Rating Inventory of Executive Functions—preschool version (BRIEF-P) [22] (Italian version [23]) has been widely used to examine the relation between Executive Functions (EF) skills and important developmental outcomes, including psychopathologies in children aged from 2 years and 0 months to 5 years and 11 months. This standardized parent-rating scale comprises 63 Likert-scale items that assess five distinct components of EF: Inhibit, Shift, Emotional Control, Working-memory, and Plan/organize. The present study focused on the scales Inhibit and Emotional Control which composes the index Inhibitory Self-Control Index (ISCI). Participants are asked to rate the child’s behavior on a 3-point scale (“Never” = 1, “Sometimes” = 2, and “Often” = 3) in terms of how often the specific behavior has been a problem. The recommended cut-off for potential clinical significance is a *T* score of 65, which represents a score of 1.5 SDs above the normative mean.

#### The Strengths and Difficulties Questionnaire—Parent version (SDQ-P)

The SDQ-P [24] (Italian version [25]), is a screening measure detecting children’ strengths and difficulties as perceived by their parents. It is a brief questionnaire comprising 25 items measured on a 3-point Likert scale (0 = ”Not True”; 1 = “Somewhat true”; 2 = “Certainly true”), including 5 subscales, namely Emotional symptoms (EMO, 5 items), Conduct problems (COND, 5 items), Hyperactivity-Inattention (HYPER, 5 items), Peer problems (PEER, 5 items) and Prosocial behaviors (PROS, 5 items), and a Total Difficulty Score (TDS).

*The Strengths and Difficulties Questionnaire*—18 + (SDQ) [24], is a screening measure detecting individual strengths and difficulties in people older than 18 years. It shows the same structure as the children’s version. For both children and mothers, we collected data only on EMO, COND, and HYPER subscales.

#### The Difficulties in Emotion Regulation (DERS)

The DERS [26] (Italian version validated by Giromini et al. [27]), is a 36-item scale developed to assess multiple facets of emotion regulation, on a 5-point Likert scale ranging from 1 (almost never) to 5 (almost always). It includes six subscales: Goal-Directed Behavior (Goals, 5 items); Impulse Control Difficulties (Impulse, 6 items); Lack of Emotional Awareness (Awareness, 6 items); Limited Access to Emotion Regulation Strategies (Strategies, 8 items); and Lack of Emotional Clarity (Clarity, 5 items).

### Statistical analysis

Mean and standard deviation (SD) of all the study variables are reported in Table 1 and the supplemental material (Tables S1, S2). The McNemar’s test was used to assess the change in the proportion of poor sleepers before and during the lockdown.

**Table 1:** Demographics and descriptive information of the mothers and their children for each working condition

			Regular working (*N* = 30)	Not working (*N* = 52)	Stopped working (*N* = 64)	Smart working (*N* = 99)
Mothers			Mean (SD)	Mean (SD)	Mean (SD)	Mean (SD)
Age (years)			38.33 (3.69)	36.59 (4.37)	35.73 (4.90)	38.43 (4.49)
Number of children in the family			1.6 (0.7)	1.7 (0.8)	1.6 (0.6)	1.7 (0.7)
Educational level (years)			15.4 (3.14)	14.5 (3.72)	14.8 (3.27)	17.3 (2.90)
DERS Total Score			79.13 (13.62)	75.10 (16.61)	75.14 (15.14)	77.32 (18.06)
COVID-19 items						
Do you fear getting infected (0–3)?			1.73 (0.69)	1.87 (0.69)	1.89 (0.65)	1.58 (0.70)
Do you know someone who has tested positive for COVID-19?			*N* (%)	*N* (%)	*N* (%)	*N* (%)
Relatives						
Deceased			6 (20.00)	8 (15.38)	16 (25.00)	16 (16.16)
Infected			1 (3.30)	2 (3.85)	5 (7.80)	4 (4.00)
Acquaintance						
Infected			19 (63.30)	32 (61.54)	47 (73.44)	68 (68.69)
Deceased			12 (40.00)	16 (30.77)	30 (48.88)	33 (33.33)
Friend						
Infected			5 (16.68)	16 (30.80)	26 (40.63)	20 (20.20)
Deceased			0 (0.00)	4 (7.70)	6 (9.38)	7 (7.10)
Children			Mean (SD)	Mean (SD)	Mean (SD)	Mean (SD)
Age (years)			4.07 (0.90)	4.02 (1.00)	4.09 (0.90)	4.16 (0.90)
			*N* (%)	*N* (%)	*N* (%)	*N* (%)
Gender (M)			19 (63.3)	28 (53.8)	38 (43.8)	53 (53.50)
Going to kindergarten			29 (96.7)	45 (86.5)	62 (98.9)	96 (97)
Informed about COVID			28 (93.30)	50 (96.10)	61 (95.30)	97 (98.00)
Informed by family			27 (90.00)	50 (96.20)	60 (93.80)	96 (97.00)
Method used						
Fables			10 (33.30)	16 (30.80)	29 (45.30)	42 (42.40)
Video			19 (63.30)	24 (46.20)	40 (62.50)	59 (59.60)
Science			7 (23.30)	11 (21.20)	11 (17.20)	23 (23.20)

For both mothers and children, a series of 4 × 2 repeated measure ANOVA with working condition (regular working, smart working, stopped working, and not working) and period (before the lockdown, during the lockdown) as within-subjects’ factors were used to assess the change in our main variables of interest. The Tukey HSD test was used for post-hoc comparisons, and partial eta squared ($\eta_{p}^{2}$) was reported as an estimate of effect size. Exploratory Pearson correlations were run to investigate the relationship between changes in sleep variables, time experience, emotional and behavioral symptoms.

To assess whether changes in both mothers and children’ routines and emotional symptoms affected children’s behaviors we built a multiple linear regression model with the change in the BRIEF-P Inhibitory Self-Control Index (ΔISCI) as the dependent variable and, as covariates: (1) children age, (2) changes in sleep quality in children (ΔSDSC), (3) changes in sleep quality in mothers (ΔPSQI), (4) change in the mothers’ SDQ scores, (5) changes in mothers’ time pressure (ΔTP), (6) fear of contagion of the mothers (7) emotional regulation ability of the mothers (DERS total score), and working condition as factor.

Another multiple linear regression was conducted with the change in the mothers’ SDQ scores as the dependent variable and: (1) children age, (2) ΔSDCQ, (3) ΔPSQI, (4) ΔISCI, (5) ΔTP, (6) fear of contagion of the mothers (7) DERS total score as covariates, and working condition as factor. Basic assumptions of linear regressions were checked computing Tolerance and VIF (for collinearity assumption), Durbin–Watson test (for assessing independent errors), and visually exploration of *Q*–*Q* plots.

For all the analyses, the level of significance was set at *p* < 0.05 and, due to the exploratory nature of this study, no correction was applied for multiple comparisons.

## Results

### Descriptive statistics

Most of the mothers (66.5%) had to stop working or to start working from home in smart modality (Table 1). Mothers had similar age and number of children regardless of working conditions. Their children had similar age, about 4 years old on average. Mothers had a relatively high fear of contagion, and most of them knew someone who had contracted COVID-19 or passed away due to the contagion. Most of the children used to go to kindergarten before the lockdown, and they were informed about COVID-19 by their family using different modalities.

### Changes in mothers

**Fig. 1:**
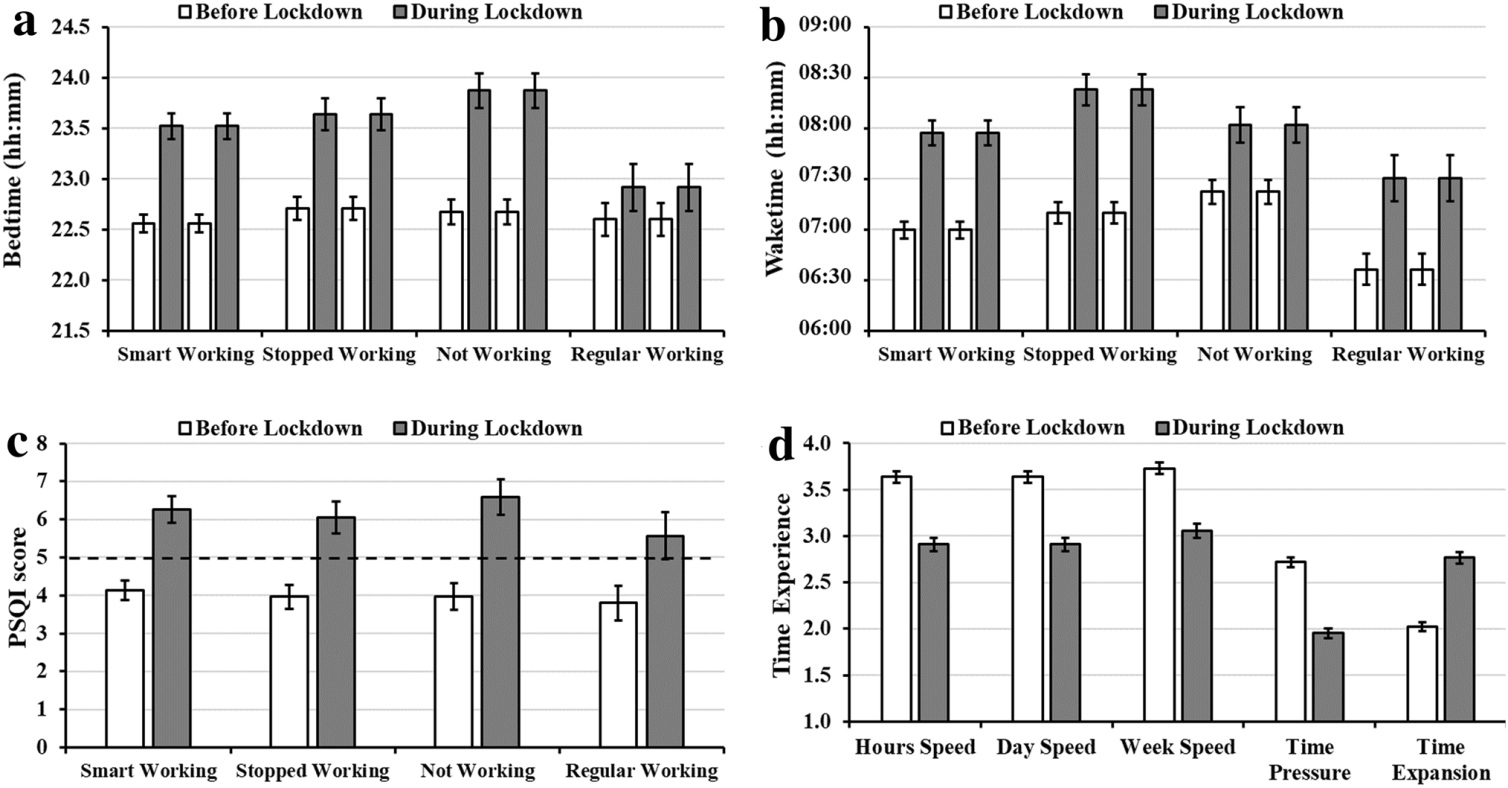
Changes in mothers’ **a** sleep quality (PSQI total score), **b** bedtime, **c** waketime, and **d** time experience as a function of the presence of the lockdown and the mothers’ working condition. For panel a, the dashed line represents the cut-off for identifying good (PSQI ≤ 5) and poor sleepers (PSQI > 5). Error bars represent standard errors

Sleep timing was markedly affected by the lockdown, with mothers starting to go bed on average ~ 54 min later (*F*_1,241_ = 116.7, *p* < 0.0001, $\eta_{p}^{2}$ = 0.33), although this effect was less marked in mothers there were still going to work outside to their home (*F*_3,242_ = 4.00, *p* = 0.009, $\eta_{p}^{2}$ = 0.04, Fig. 1a). Mothers started to wake up ~ 57 min later (*F*_1,241_ = 129.89, *p* < 0.0001, $\eta_{p}^{2}$ = 0.35, Fig. 1b), regardless of the working situation. Overall, mothers showed a sleep quality worsening during lockdown (*F*_1,241_ = 76.91, *p* < 0.0001, $\eta_{p}^{2}$ = 0.24, Fig. 1c), regardless of the working situation. This worsening was also confirmed by the proportion of mothers reporting poor sleep (i.e., PSQI > 5), which increased from 21.54% before the lockdown to 48.37% during the lockdown ($\chi_{1}^{2}$ = 42.7, *p* < 0.001; odds ratio 4.82 [CI 2.84–8.68]).

Focusing on time experience (Fig. 1d), we observed that the mothers felt that the speed of the hours (*F*_1,241_ = 56.07, *p* < 0.0001, $\eta_{p}^{2}$ = 0.19), the days (*F*_1,241_ = 60.26, *p* < 0.0001, $\eta_{p}^{2}$ = 0.20), and the week (*F*_1,241_ = 46.56, *p* < 0.0001, $\eta_{p}^{2}$ = 0.16) were slowing down during the lockdown. Moreover, they felt a marked decrease in time pressure (*F*_1,241_ = 135.78, *p* < 0.0001, $\eta_{p}^{2}$ = 0.36) and an increase in time expansion (*F*_1,241_ = 116.13, *p* < 0.0001, $\eta_{p}^{2}$ = 0.33) during the lockdown.

Looking at the SDQ subscales, we observed an increase in emotion symptoms (EMO) during the lockdown (*F*_1,241_ = 19.41, *p* < 0.0001, $\eta_{p}^{2}$ = 0.07), but not in conduct problems (COND) or hyperactivity/inattention (HYPER) (all *p*’s > 0.23).

### Changes in children

**Fig. 2:**
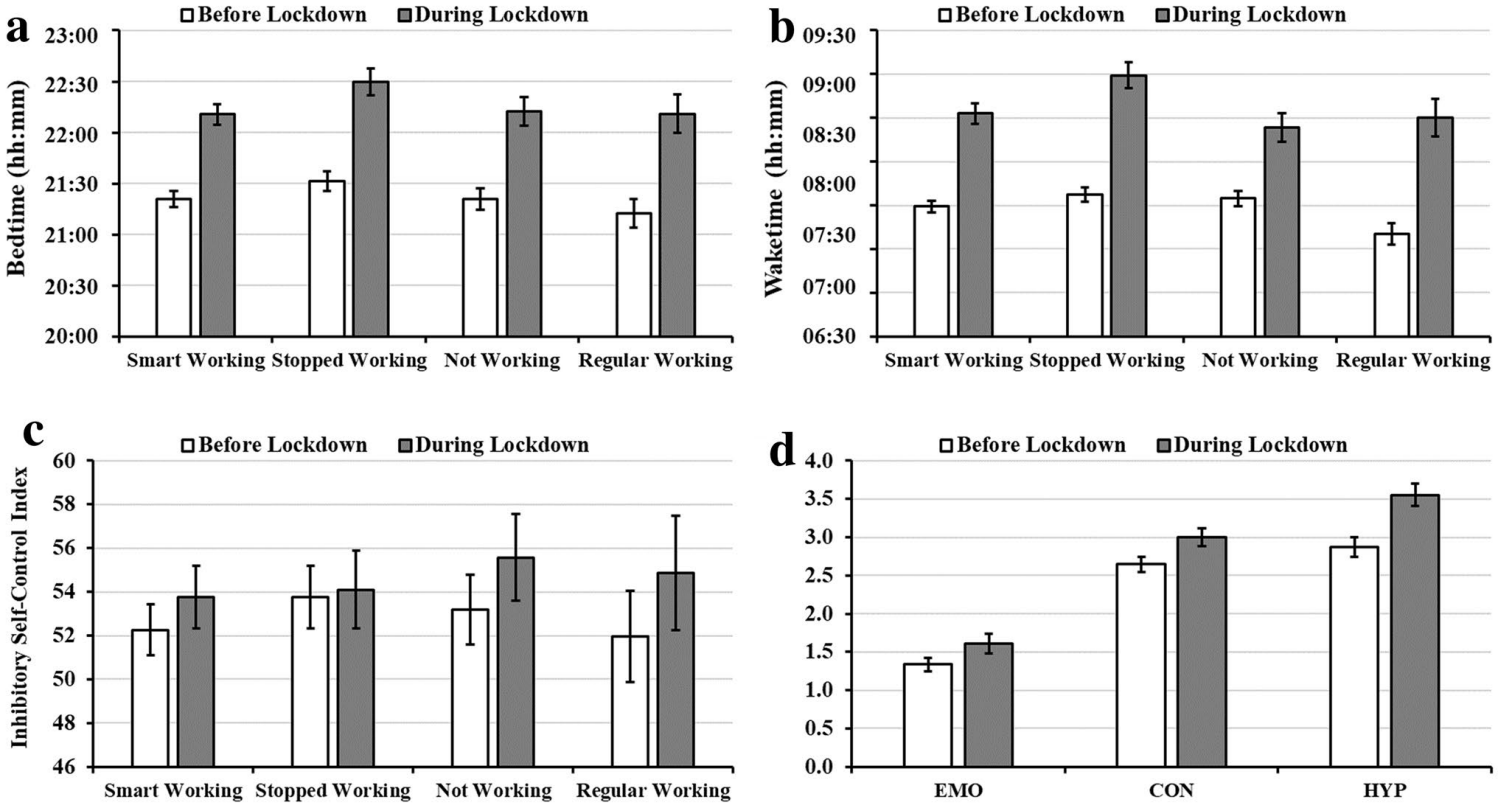
Changes in children’s **a** bedtime, **b** waketime, **c** inhibitory self-control (ISCI), and **d** SDQ subscales as a function of the presence of the lockdown and the mothers’ working condition. For panel *EMO* emotional symptoms subscale, *CON* conduct problems subscale, *HYP* hyperactivity/inattention subscale. Error bars represent standard errors

As for the mothers, children’ sleep timing was markedly affected by the lockdown. Children went to be on average ~ 53 min later (*F*_1,241_ = 259.0, *p* < 0.0001, $\eta_{p}^{2}$ = 0.52, Fig. 2a), and wake up ~ 66 min later (*F*_1,241_ = 260.35, *p* < 0.0001, $\eta_{p}^{2}$ = 0.52, Fig. 2b). The total score of the SDSC did not significantly change during the lockdown (*F*_1,241_ = 0.001, *p* = 0.970, $\eta_{p}^{2}$ = 0.01). Similarly, the proportion of children with some sleep difficulties (i.e., SDSC > 39) was stable, from 41.46% before the lockdown to 44.72% during the lockdown ($\chi_{1}^{2}$ = 0.71, *p* = 0.399; odds ratio 1.23 [CI 0.74–2.04]).

As regards time experience, mothers reported an increased sense of boredom during the lockdown in their children (*F*_1,241_ = 79.39, *p* < 0.0001, $\eta_{p}^{2}$ = 0.24), in particular mothers who kept working in smart modality. Children also showed increased difficulties to follow daily routines (*F*_1,241_ = 55.35, *p* < 0.0001, $\eta_{p}^{2}$ = 0.19), although their ability to keep track of the passage of time did not change during the lockdown (*F*_1,241_ = 0.01, *p* = 0.938, $\eta_{p}^{2}$ < 0.01).

Focusing on the BRIEF-P, children showed an increased score in the Inhibitory Self-Control Index (ISCI) (*F*_1,241_ = 7.43, *p* = 0.007, $\eta_{p}^{2}$ = 0.03, Fig. 2c), with the proportion of children with self-control difficulties (i.e., ISCI > 65) increased from 14.29% before the lockdown to 21.23% during the lockdown ($\chi_{1}^{2}$ = 12.6, *p* < 0.001; odds ratio 6.67 [CI 1.98–35.04]).

Looking at the SDQ subscales, we observed an increase in emotion symptoms (EMO) (*F*_1,241_ = 6.57, *p* = 0.011, $\eta_{p}^{2}$ = 0.03), conduct problems (COND) (*F*_1,241_ = 9.01, *p* = 0.003, $\eta_{p}^{2}$ = 0.04), and hyperactivity/inattention (HYPER) issues (*F*_1,241_ = 31.56, *p* < 0.0001, $\eta_{p}^{2}$ = 0.12) during the lockdown, regardless of the mother working situation (Fig. 2d).

### Relationship between mothers’ and children’ sleep changes

**Fig. 3:**
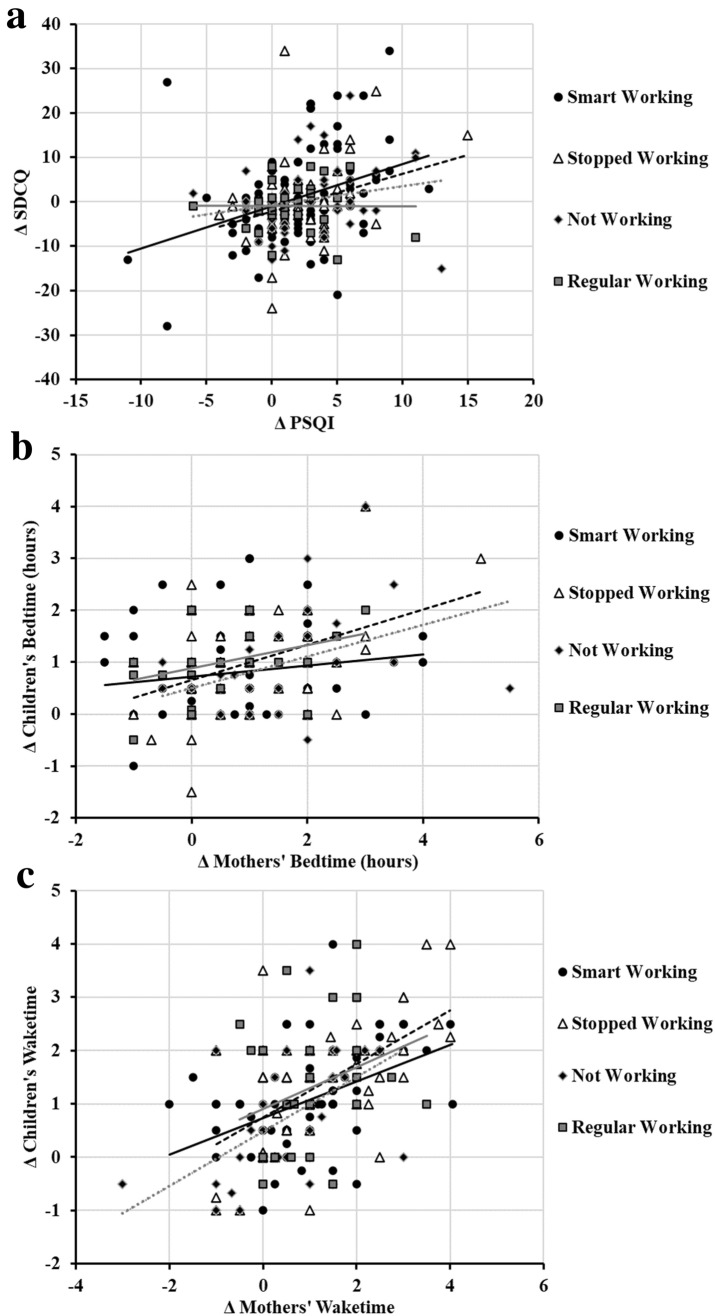
Relationship between changes in children and mother **a** sleep quality (ΔSDCQ and ΔPSQI respectively), **b** bedtime, and **c** waketime. Note that higher scores in ΔSDCQ and ΔPSQI indicate lower sleep quality during lockdown compared to the period before the lockdown

Considering the age of the children (2–5 years old), it was expected that changes in the mothers' sleep timing would be associated with those of their children. Moreover, we expected that a lowering in children’ sleep quality would make their mothers’ sleep worse. Exploring these relationships, we observed a positive association between changes in the sleep quality of mothers and their children (*r* = 0.25, *p* < 0.001, Fig. 3a), although this relationship was driven by the mothers who continued working from home (*r* = 0.29, *p* = 0.003), and by the mothers who used to work but had to stop during the lockdown (*r* = 0.31, *p* = 0.012), but not by the mothers who did not work (*r* = 0.21, *p* = 0.135), and by the mother who continued working outside (*r* = − 0.10, *p* = 0.590). Also, later the mothers went to bed during the lockdown, later did their children (*r* = 0.30, *p* < 0.001), again with some differences due to the working conditions (see Fig. 3b). Similarly, the change in mother waketime was strongly associated with the change in children’ waketime (*r* = − 0.50, *p* < 0.001, Fig. 3c).

### Factors associated with change in inhibitory self-control in children

The multiple regression model showed that the changes in children’s inhibitory self-control were associated with their sleep quality as well as their mothers’ sleep quality, i.e., worst sleep quality, worse inhibitory self-control, and by the total score of the mothers’ DERS, i.e., more the mothers had difficulties in regulating emotions, worse resulted the children’s inhibitory self-control (see Table 2). Children’ age, working condition of mothers, and the other factors were not significant associated with the changes in children’s inhibitory self-control. For all variables, tolerance was > 0.872 and VIF was < 1.14, indicating that multicollinearity was not a concern. Also, the data met the assumption of independent errors (Durbin–Watson value = 1.86) and the normal *Q*–*Q* plot of standardized residuals showed points that were not completely on the line, but close.

**Table 2:** Multiple regressions on change in inhibitory self-control in children

	Δ Inhibitory self-control					
	*F*	*df*	*b* (95% CI)	Std. *β*	*t*	*p*
Intercept			− 1.75 (− 9.50, 6.00)	0	− 0.45	0.657
Children’s Age	32.7	1	0.40 (− 0.77, 1.64)	0.04	0.67	0.499
Δ SDSC	**30.46**	**1**	**0.38 (0.24, 0.51)**	**0.34**	**5.52**	**< 0.001**
Δ PSQI	**6.63**	**1**	**0.45 (0.10, 0.79)**	**0.16**	**2.15**	**0.011**
Mothers’ ΔTP	1.51	1	0.73 (− 0.44, 1.89)	0.07	1.23	0.221
Mothers’ ΔSDQ	2.41	1	0.25 (− 0.07, 0.57)	0.10	1.55	0.122
Mothers’ DERS	**4.63**	**1**	**0.07 (0.01, 0.14)**	**0.13**	**2.15**	**0.032**
Mothers’ Fear of COVID-19	3.30	1	− 1.54 (− 3.22, 0.13)	− 0.11	− 1.82	0.071
Working condition	1.04	3				0.374
Not working^§^			− 1.41 (− 5.29, 2.47)	− 0.15	− 0.72	0.474
Stopped working^a^			− 2.66 (− 6.39, 1.07)	− 0.28	− 1.41	0.161
Smart working^a^			− 2.84 (− 6.34, 0.67)	− 0.30	− 1.60	0.112
Model fit			*F* (10, 234) = 7.72 *p* < 0.001			
Adj. *R*^2^			0.216			

*b* unstandardized beta, *std*. *β* standardized beta, *CI* confidence intervals, *ΔSDSC* change in Sleep Disturbance Scale for Children, *DERS* Difficulties in Emotion Regulation Scale, *ΔPSQI* change in the Pittsburgh Sleep Quality Index, *ΔTP* change in time pressure, *ΔSDQ* changes in Strength and Difficulties questionnaire total score

^a^Reference: regular workers

### Factors associated with change in SDQ total scores in mothers

The multiple regression on the change in mothers' strengths and difficulties (Table 3) showed the change in their sleep quality as well as in their children was associated with the change in their SDQ total score (i.e., worst the sleep quality, worse the SDQ scores). Also, the change in time pressure was associated with the change in SDQ total score (i.e., increased time pressure, worse SDQ scores) and in the fear of the COVID-19 (higher the fear, worse the SDQ scores). The other factors, including the working condition, were not significant. Variables showed a tolerance > 0.879 and a VIF < 1.15, indicating again that multicollinearity was not a concern. The data met the assumption of independent errors (Durbin–Watson value = 2.02) and the normal *Q*–*Q* plot of standardized residuals showed points that were not completely on the line, but close.

**Table 3:** Multiple regressions on change in SDQ total score in mothers

	Δ Strength and difficulties					
	*F*	*df*	*b* (95% CI)	Std. *β*	*t*	*p*
Intercept			− 1.24 (− 4.35, 1.88)	0	− 0.78	0.434
Δ PSQI	**26.26**	**1**	**0.34 (0.21, 0.48)**	**0.32**	**5.12**	**< 0.001**
Mothers’ ΔTP	**3.92**	**1**	**0.47 (0.01, 0.93)**	**0.12**	**1.98**	**0.049**
Mothers’ DERS	1.44	1	− 0.02 (− 0.05, 0.01)	− 0.07	− 1.20	0.231
Mothers’ Fear of COVID-19	**4.40**	**1**	**0.71 (0.04, 1.39)**	**0.13**	**1.39**	**0.037**
Children’s Age	1.03	1	0.24 (− 0.23, 0.71)	0.06	1.01	0.312
Children’s ΔISCI	2.41	1	0.04 (− 0.01, 0.09)	0.10	1.55	0.122
Δ SDSC	**6.29**	**1**	**0.07 (0.02, 0.13)**	**0.16**	**2.51**	**0.013**
Working condition	1.24	3				0.296
Not working^§^			0.25 (− 1.32, 1.80)	0.07	0.31	0.755
Stopped working^a^			1.13 (− 0.37, 2.63)	0.30	1.49	0.139
Smart working^a^			0.98 (− 0.43, 2.39)	0.29	1.37	0.172
Model fit			*F* (10, 234) = 7.17 *p* < 0.001			
Adj. *R*^2^			0.202			

*b* unstandardized beta, std. *β* standardized beta, *CI* confidence intervals, *ΔSDSC* change in Sleep Disturbance Scale for Children, *DERS* Difficulties in Emotion Regulation Scale, *ΔPSQI* change in the Pittsburgh Sleep Quality Index, *ΔTP* change in time pressure, *ΔISCI* changes in Inhibitory Self-Control in children

^a^Reference: regular workers

## Discussion

The present research was aimed at investigating how home confinement and the related social restrictions imposed in Italy by March 10th impacted mothers and their pre-school children's behavioral habits and psychological well-being. As expected, data showed that these restrictive measures had negative effects on mothers’ and their children’s behavioral and psychological levels, with some differences depending on the mothers working situation, in line with previous studies [2]. Broadly speaking, it seemed that smart working modality or unexpected working interruption impacted negatively on habits and quality of sleep.

Mothers' sleep quality, timing, and feelings about time flow markedly changed, consistently with a recent study in another Italian sample under lockdown [8]. Children' sleep quality was, on average, less affected by the lockdown, although their sleep timing strongly shifted (they went to bed ~ 53 min and woke up ~ 66 min later than usual). These changes, together with reported difficulties of following routines, indicate a substantial breakdown in their daily routines. This aspect is particularly relevant because one of the most protective factors in preserving both physical and psychological well-being in children is having a structured and pre-planned day [13].

As for the psychological functioning, concerning the period before quarantine, mothers reported an increasing level of emotional symptoms such as sadness and frustration, whereas they perceived their children also more undisciplined and hyperactive, with a worsening inhibitory self-control capacity. Children's psychological outcomes could be considered as the result of a breakdown in their daily routines, impossibility to discharge their physical energy due to the home confinement, and the temporary interruption of their peer relationship, considering that most of them attended kindergarten [28]. These results are in line with previous studies that showed an increase in emotional disturbance and exhaustion, low mood, and irritability in parents and children in quarantine [5, 12, 29].

When the interplay between the behavioral and psychological factors was investigated, the factor that seems to mostly impact both mothers' and children's psychological well-being was their sleep quality, as recently predicted by the task force of the European CBT-I Academy [14]. That is, not only children's sleep quality but also that of their mothers were associated with children's ability to regulate and control their behavior. A similar result was observed for mothers' strengths and difficulties.

Besides their and their mothers’ sleep quality, children inhibitory self-control capacity seemed to be also associated with mothers’ trait emotional fatigue. This result can be interpreted in two ways. Mothers’ difficulties in emotion regulation may have affected children’s behaviors, but it also possible that mothers’ trait emotional distress may have influenced the perception that they have about their children. Since the emotional regulation of mothers has a key role in children’s psychological development [30], this aspect deserves to be considered when psychological support programs will be implemented after the lockdown and for a future emergency scenario involving families.

Concerning the mothers, their strengths and difficulties seemed to be associated not only with their sleep quality and that of their children, but also with the fear of COVID-19, and with the time pressure they felt. This is due probably to the fact that mothers during the lockdown need to conciliate time for their children, house-holding, and some of them also for personal work, so time pressure seems to impact their distress. All these factors are likely related in a bidirectional fashion with difficult night-time [14].

Although the present study has attempted to provide a snapshot as realistic as possible of the current Italian situation, it is not without limits. First, our small sample size could have decreased our statistical power, limiting the significance of some of the statistical comparisons conducted. Therefore our sample cannot be considered representative of all the mothers of the Italian population. Second, and related to the previous point, even if some mothers who took part in the research were involved by word of mouth or because they were part of parent groups of children who attend to the same kindergartens, our sample is mainly composed of mothers who were seeking information on research-related websites and social media groups. Therefore, future study should consider, among the other factors involved, also this bias that concerns how participants learned or are informed of research. Third, the absence of error rate correction, due to the exploratory nature of this study. Four, the research focused on mothers, as they are probably the primary caregivers for children within the age group considered here. Of note, of the total sample, only 7 mothers declared that they were alone in raising their child. This implies that in future studies it will be necessary to take into consideration not only the mother but also other figures, such as the other parent, that are directly involved to raise children. Further, caregivers’ perception about the support they received in the care and management of their child would be useful to address. Finally, the present research, as other recent studies on the COVID-19 pandemic [8, 31], used retrospective questions to compare the current situation to a baseline before the outbreak. Although some pitfalls and biases associated with this method may be present, it has been recently demonstrated that data elicited by retrospective questions are quite consistent [32], especially, when there have been no changes between the current answers at all times, as in the present research.

## Conclusion

Overall, this data indicates that the Italian total lockdown, which involved the closure of schools and individual home confinement, is particularly challenging for mothers and their children, given the reliance on carefully established daily-routines and relationships, as well as informal support in childcare. These data are particularly relevant in Italy, as the Government is moving from a total to a partial lockdown, with the reopening of all shops, and services, but with the indication of using smart working whenever possible. However, it has not foreseen yet the reopening of the schools. Moreover, the Government should also plan special programs for families, including not only psychological support but specific projects to sustain families with working parents in ameliorating children's management. Besides Italy, these results may be important for developing policies in several countries that are facing the COVID-19 outbreak. Regardless of the welfare system and the cultural differences, mothers’ and their children's well-being need to be taken seriously, and economic as well as psychosocial interventions are urgently warranted.

## Electronic supplementary material

Below is the link to the electronic supplementary material.

Supplementary file1 (DOCX 24 kb)

## Data Availability

The data that support the findings of this study are available from the corresponding author upon reasonable request.
