# Supplementary material for: The interplay between mothers’ and children behavioral and psychological factors during COVID-19: an Italian study
Source: Eur Child Adolesc Psychiatry. 2020 Aug 31;30(9):1401–12. doi: 10.1007/s00787-020-01631-3 (PMC7456665; doi:10.1007/s00787-020-01631-3)
Supplement: Supplementary file 1 — Supplementary file1 (DOCX 24 kb) [file 787_2020_1631_MOESM1_ESM.docx]

**SUPPLEMENTARY MATERIAL**

Ms ID: ECAP-D-20-00222,

European Child & Adolescent Psychiatry

Title: ***The interplay between mothers’ and children behavioral and psychological factors during COVID-19: An Italian study***

by Di Giorgio (surname), E., Di Riso (surname), D., Mioni, G., & Cellini, N.

**Table S1.** Mean and standard deviation (SD) of the variables before and during the lockdown in the mothers.

| \|  \| **Regular Working** (N=30) \| **Not Working** (N=52) \| **Stopped Working** (N=64) \| **Smart Working** (N=99) \| \| --- \| --- \| --- \| --- \| --- \| \| **MOTHERS** \| **Mean (SD)** \| **Mean (SD)** \| **Mean (SD)** \| **Mean (SD)** \| \| ***Sleep variables*** \|  \|  \|  \|  \| \| *PSQI Before the lockdown* \| 3.80 (2.58) \| 3.96 (2.27) \| 3.97 (2.24) \| 4.14 (2.82) \| \| *PSQI During the lockdown* \| 5.56 (3.47) \| 6.60 (3.56) \| 6.06 (3.09) \| 6.26 (3.47) \| \| *Bedtime Before the lockdown (hh:mm)* \| 22:34 (00:05) \| 22:43 (00:07) \| 22:40 (00:08) \| 22:36 (00:10) \| \| *Bedtime During the lockdown (hh:mm)* \| 23:31 (00:08) \| 23:39 (00:09) \| 22:52 (00:10) \| 22:55 (00:14) \| \| *Waketime Before the lockdown (hh:mm)* \| 07:00 (00:05) \| 07:09 (00:06) \| 07:22 (00:07) \| 06:36 (00:09) \| \| *Waketime During the lockdown (hh:mm)* \| 07:57 (00:09) \| 08:22 (00:07) \| 08:02 (00:10) \| 07:30 (00:14) \| \| ***Strength and Difficulties*** \|  \|  \|  \|  \| \| *EMO Before the lockdown* \| 3.27 (2.21) \| 2.27 (2.55) \| 2.80 (2.05) \| 2.61 (2.20) \| \| *EMO During the lockdown* \| 3.60 (2.04) \| 3.50 (2.48) \| 3.70 (2.47) \| 3.26 (2.63) \| \| *COND Before the lockdown* \| 1.87 (1.14) \| 1.98 (1.41) \| 1.69 (1.14) \| 1.91 (1.32) \| \| *COND During the lockdown* \| 1.73 (1.20) \| 1.94 (1.36) \| 1.89 (1.22) \| 2.03 (1.42) \| \| *HYPER Before the lockdown* \| 4.17 (1.53) \| 4.31 (1.65) \| 3.91 (1.39) \| 4.17 (1.45) \| \| *HYPER During the lockdown* \| 3.87 (1.33) \| 4.39 (1.56) \| 3.98 (1.63) \| 4.43 (1.63) \| \| *SDQ Before the lockdown* \| 9.30 (4.12) \| 9.00 (4.45) \| 8.39 (3.17) \| 3.27 (2.12) \| \| *SDQ During the lockdown* \| 9.20 (3.34) \| 9.83 (4.25) \| 9.58 (4.02) \| 9.73 (4.66) \| \| ***Time Experience*** \|  \|  \|  \|  \| \| *Hour speed Before the lockdown* \| 3.67 (0.94) \| 3.50 (0.80) \| 3.56 (0.73) \| 3.76 (0.89) \| \| *Hour speed during the lockdown* \| 3.03 (0.81) \| 3.02 (0.94) \| 2.75 (1.05) \| 2.96 (1.03) \| \| *Day speed before the lockdown* \| 3.60 (1.00) \| 3.52 (0.83) \| 3.63 (0.77) \| 3.81 (0.97) \| \| *Day speed during the lockdown* \| 3.03 (1.03) \| 2.96 (0.91) \| 2.67 (1.05) \| 2.96 (1.10) \| \| *Week speed before the lockdown* \| 3.67 (0.96) \| 3.69 (0.83) \| 3.73 (0.83) \| 3.87 (0.94) \| \| *Week speed during the lockdown* \| 3.30 (1.02) \| 3.12 (1.11) \| 2.70 (1.08) \| 3.11 (1.11) \| \| *TP before the lockdown* \| 2.76 (0.77) \| 2.45 (0.67) \| 2.72 (0.67) \| 2.95 (0.76) \| \| *TP during the lockdown* \| 1.96 (0.79) \| 2.00 (0.84) \| 1.72 (0.55) \| 2.14 (0.78) \| \| *TE before the lockdown* \| 1.66 (0.48) \| 2.03 (0.74) \| 2.14 (0.66) \| 1.99 (0.62) \| \| *TE during the lockdown* \| 2.35 (0.75) \| 3.01 (0.93) \| 3.03 (0.90) \| 2.67 (0.89) \| |  | | |  |  |  |  |
| --- | --- | --- | --- | --- | --- | --- | --- | --- | --- | --- | --- | --- | --- | --- | --- | --- | --- | --- | --- | --- | --- | --- | --- | --- | --- | --- | --- | --- | --- | --- | --- | --- | --- | --- | --- | --- | --- | --- | --- | --- | --- | --- | --- | --- | --- | --- | --- | --- | --- | --- | --- | --- | --- | --- | --- | --- | --- | --- | --- | --- | --- | --- | --- | --- | --- | --- | --- | --- | --- | --- | --- | --- | --- | --- | --- | --- | --- | --- | --- | --- | --- | --- | --- | --- | --- | --- | --- | --- | --- | --- | --- | --- | --- | --- | --- | --- | --- | --- | --- | --- | --- | --- | --- | --- | --- | --- | --- | --- | --- | --- | --- | --- | --- | --- | --- | --- | --- | --- | --- | --- | --- | --- | --- | --- | --- | --- | --- | --- | --- | --- | --- | --- | --- | --- | --- | --- | --- | --- | --- | --- | --- | --- | --- | --- | --- | --- | --- | --- | --- | --- | --- | --- |
|  |  | |  |  |  |  |  |
| **Notes**. PSQI: Pittsburgh Sleep Quality Index total score. EMO: Emotional symptoms. COND: Conduct problems. HYPER: Hyperactivity-Inattention problems. SDQ: Strength and Difficulties total score. TP: Time Pressure. TE: time Expansion | |  | |  |  |  |  |

**Table S2.** Mean and standard deviation (SD) of the study variables before and during the lockdown in the children.

|  | **Regular Working** (N=30) | **Not Working** (N=52) | **Stopped Working** (N=64) | **Smart Working** (N=99) |
| --- | --- | --- | --- | --- |
| **CHILDREN** | **Mean (SD)** | **Mean (SD)** | **Mean (SD)** | **Mean (SD)** |
| ***Sleep variables*** |  |  |  |  |
| *SDSC Before the lockdown* | 40.93 (9.03) | 41.35 (12.18) | 39.69 (8.64) | 39.49 (8.24) |
| *SDSC During the lockdown* | 39.70 (8.65) | 41.65 (11.64) | 39.30 (8.64) | 40.50 (10.56) |
| *Bedtime Before the lockdown (hh:mm)* | 21:20 (00:05) | 21:31 (00:06) | 21:22 (00:06) | 21:12 (00:10) |
| *Bedtime During the lockdown (hh:mm)* | 22:10 (00:06) | 22:30 (00:08) | 22:12 (00:08) | 22:11 (00:11) |
| *Waketime Before the lockdown (hh:mm)* | 07:29 (00:04) | 07:38 (00:05) | 07:35 (00:05) | 07:11 (00:07) |
| *Waketime During the lockdown (hh:mm)* | 08:33 (00:07) | 08:59 (00:09) | 08:23 (00:10) | 08:30 (00:13) |
| ***Strength and Difficulties*** |  |  |  |  |
| *EMO Before the lockdown* | 1.53 (1.17) | 1.27 (1.29) | 1.38 (1.39) | 1.13 (1.29) |
| *EMO During the lockdown* | 1.80 (1.38) | 1.65 (1.78) | 1.58 (1.97) | 1.41 (1.84) |
| *COND Before the lockdown* | 2.77 (1.36) | 2.77 (1.70) | 2.50 (1.31) | 2.49 (1.16) |
| *COND During the lockdown* | 3.00 (1.80) | 3.27 (1.82) | 2.64 (1.48) | 3.07 (1.75) |
| *HYPER Before the lockdown* | 2.77 (1.38) | 2.62 (2.23) | 3.29 (1.96) | 2.79 (1.77) |
| *HYPER During the lockdown* | 3.27 (1.33) | 3.67 (2.53) | 3.77 (2.23) | 3.51 (1.90) |
| *SDQ Before the lockdown* | 6.87 (2.76) | 6.65 (3.69) | 7.16 (3.21) | 6.04 (2.64) |
| *SDQ During the lockdown* | 8.07 (3.52) | 8.60 (4.83) | 7.98 (4.41) | 7.99 (4.17) |
| ***BRIEF-P*** |  |  |  |  |
| *ISCI Before the lockdown* | 50.87 (11.22) | 51.42 (12.83) | 52.97 (13.21) | 50.64 (09.59) |
| *ISCI During the lockdown* | 54.87 (10.31) | 55.58 (16.68) | 54.09 (15.13) | 53.75 (13.33) |
| ***Time Experience*** |  |  |  |  |
| *Boredom before the lockdown* | 0.73 (0.74) | 0.67 (0.73) | 0.86 (0.69) | 0.52 (0.56) |
| *Boredom during the lockdown* | 1.17 (0.83) | 1.21 (0.90) | 1.34 (0.98) | 1.382 (0.85) |
| *Daily routinnes before the lockdown* | 0.67 (0.71) | 0.44 (0.64) | 0.50 (0.67) | 0.43 (0.67) |
| *Daily routinnes during the lockdown* | 0.87 (0.82) | 0.96 (0.97) | 1.14 (0.92) | 0.90 (0.72) |
| *Time tracking before the lockdown* | 1.00 (0.95) | 0.94 (0.90) | 1.08 (0.91) | 1.18 (1.02) |
| *Time tracking during the lockdown* | 1.33 (0.81) | 0.98 (0.92) | 1.05 (0.88) | 1.16 (0.98) |

**Notes**. SDSC: Sleep Disturbances Scale for Children total score. EMO: Emotional symptoms. COND: Conduct problems. HYPER: Hyperactivity-Inattention problems. SDQ: Strength and Difficulties total score. ISCI: Inhibitory Self-Control Index.
